# Three new species and a new combination of *Triblidium*

**DOI:** 10.3897/mycokeys.60.46645

**Published:** 2019-10-31

**Authors:** Tu Lv, Cheng-Lin Hou, Peter R. Johnston

**Affiliations:** 1 College of Life Science, Capital Normal University, Xisanhuanbeilu 105, Haidian, Beijing 100048, China Capital Normal University Beijing China; 2 Manaaki Whenua Landcare Research, Private Bag 92170, Auckland 1142, New Zealand Manaaki Whenua Landcare Research Auckland New Zealand

**Keywords:** *
Huangshania
*, phylogenetic, taxonomy, Triblidiaceae, three new taxa, muriform ascospores

## Abstract

Triblidiaceae (Rhytismatales) currently consists of two genera: *Triblidium* and *Huangshania*. *Triblidium* is the type genus and is characterised by melanized apothecia that occur scattered or in small clusters on the substratum, cleistohymenial (opening in the mesohymenial phase), inamyloid thin-walled asci and hyaline muriform ascospores. Before this study, only the type species, *Triblidium caliciiforme*, had DNA sequences in the NCBI GenBank. In this study, six specimens of *Triblidium* were collected from China and France and new ITS, mtSSU, LSU and RPB2 sequences were generated. Our molecular phylogenetic analysis and morphological study demonstrated three new species of *Triblidium*, which are formally described here: *T. hubeiense*, *T. rostriforme* and *T. yunnanense*. Additionally, our results indicated that *Huangshania* that was considered to be distinct from *Triblidium* because of its elongated, transversely-septate ascospores, is congeneric with *Triblidium*. Therefore, we have placed *Huangshania* in synonymy under *Triblidium*, rendering Triblidiaceae a monotypic family.

## Introduction

*Triblidium* Rebent.: Fr. is the type genus of Triblidiaceae Rehm (Rehm 1888−1896, 1912), which includes presumed saprobes on the bark of Pinaceae, Ericaceae and Fagaceae (Magnes 1997). In his monograph of the family, Magnes (1997) speculated that some species may exist in an endophytic state. Species of *Triblidium* are well documented in Europe, but they are poorly understood in Asia and America (Magnes 1997). Magnes (1997) revised *Triblidium* and accepted amongst the many included species only four species and one subspecies.

A history of Triblidiaceae is given in Karakehian et al. (2019). In brief, Magnes (1997) placed Triblidiaceae in Rhytismatales and treated Triblidiales as a synonym of Rhytismatales. Recent five-locus (Prieto et al. 2019) and 15-locus (Johnston et al. 2019) phylogeny analyses found high support for *Pseudographis* (Triblidiaceae) within Rhytismatales. The results of a three-gene phylogenetic analysis with expanded sampling by Karakehian et al. (2019) supported Magnes classification and the authors emended Triblidiaceae to include *Triblidium* and *Huangshania*.

We conducted a morphological analysis of a specimen of *T. caliciiforme* Rebent.: Fr., the type species of *Triblidium* and additional collections of Triblidiaceae. Phylogenetic relationships were inferred based on internal transcribed spacer (ITS), nuclear large subunit ribosomal DNA (LSU), mitochondrial small subunit ribosomal DNA (mtSSU) and the second largest subunit of RNA polymerase II (RPB2) gene.

## Materials and methods

### Morphological studies and isolation

A specimen of *Triblidium caliciiforme* was collected in France in June 2012 on *Quercus* sp. Other specimens were collected in China between 2006 and 2018. Mature dried ascomata were selected for morphological observation. All observations were made from dead herbarium material. Gross morphology was observed and photographed with a dissecting microscope (Nikon SMZ-1000). Standardised colour values matching the colour of the hymenium were taken from https://www.colorhexa.com/. Microscopic preparations were observed in distilled water, Lugol’s solution (IKI), 5% potassium hydroxide (KOH) and lactophenol solution. Methods for morphological analysis follow Hou et al. (2009). Measurements of asci and ascospores were made in distilled water in 2019. For each structure, at least 25 measurements were recorded. Microphotographs were obtained using an Olympus BX51 compound microscope. Specimens are deposited in the Herbarium of the College of Life Science, Capital Normal University, Beijing, China (**BJTC**). Fresh specimens were used to obtain cultures directly from single ascoma, after washing and surface sterilisation, as follows: 75% ethanol for 10 s, 10% sodium hypochlorite for 3 min, washing in sterile water three times. The single ascoma was dried in sterilised tissue paper, placed on potato dextrose agar (PDA) with 50 mg/l chloramphenicol and incubated at room temperature (25 °C ± 3 °C). We were unable to obtain cultures from ascomata after a month.

### DNA extraction and PCR amplification

Genomic DNA was extracted from ascomata using NuClean Plant Genomic DNA Kit (CWBIO, China), following the manufacturer’s instructions and stored at -20 °C. Sequences of ITS, LSU, mtSSU and RPB2 were obtained. PCR amplifications were undertaken using primers ITS1F/ITS4 for ITS, mrSSU1/mrSSU3R for mtSSU, LR0R/LR5 for LSU and 5F/7CR for RPB2 (Vigalys and Hester 1990, White et al. 1990, Gardes and Bruns 1993, Rehner and Samuels 1994, Liu et al. 1999, Zoller et al. 1999). ITS, mtSSU and LSU PCR procedures in 25 µl reactions were carried out as outlined by Hou et al. (2009). PCR amplification of the RPB2 region was undertaken with an initial denaturation at 95 °C for 5 min, followed by 35 cycles of denaturation at 95 °C for 60 s, annealing at 55 °C for 60 s and elongation at 72 °C for 2 min and a final elongation at 72 °C for 10 min (Liu et al. 1999). The PCR products were purified, sequenced and edited by ZhongKe Xilin Biotechnology Co., Ltd. (Beijing, China). The new sequences were submitted to the NCBI GenBank database. Their accession numbers, as well as those for other ITS, LSU, mtSSU and RPB2 sequences downloaded from GenBank, are given in Table 1.

**Table 1.** Species and GenBank accession numbers of the sequences analysed in this study. “−” indicates data unavailable. Sequences generated for this study are in boldface.

**Species**	**Voucher and strain**	**ITS**	**LSU**	**mtSSU**	**RPB2**
*Bisporella citrina*	AFTOL-ID 1301	−	FJ176871	FJ190632	FJ238354
*Coccomyces dentatus*	AFTOL-ID 147	DQ491499	AY544657	AY544736	DQ247789
*Coccomyces lauraceus*	ICMP:18319	−	HM140504	HM143781	−
*Coccomyces tumidus*	Lantz 396 (UPS)	−	HM140510	HM143787	−
*Colpoma quercinum*	Lantz 368 (UPS)	−	HM140513	HM143789	−
*Cryptomyces maximus*	Lantz & Minter 424 (UPS)	−	HM140514	HM143790	−
*Cudonia circinans*	Lantz & Widen 402 (UPS)	−	HM140515	HM143791	−
*Huangshania verrucosa*	UME-29336a	MK751793	MK751802	MK751716	−
*Hypoderma rubi*	ICMP:17339	JF683419	HM140526	HM143801	−
*Hypohelion scirpinum*	Lantz 394 (UPS)	−	HM140531	HM143806	−
*Lirula macrospora*	Isolate 13	HQ902159	HQ902152	−	−
*Lophodermium eucalypti*	ICMP:16796	EF191235	HM140541	HM143817	−
*Neofabraea malicorticis*	AFTOL-ID 149	−	AY544662	AY544751	−
*Pseudographis elatina*	GJO-0090016	MK751794	MK751803	MK751717	−
*Pseudographis pinicola*	FH-18061706	MK751795	MK751804	MK751718	−
	FH-NB842	MK751796	MK751805	MK751719	−
*Sporomega degenerans*	Lantz 367 (UPS)	−	HM140567	HM143839	−
*Spathularia flavida*	KUS-F52331	JN033405	JN086708	JN086781	JN086859
*Therrya abieticola*	HOU447A	KP322574	KP322579	KP322587	−
*Triblidium caliciiforme*	FH-15071105	MK751797	MK751806	MK751720	−
	CUP-18080101	MK751798	MK751807	MK751721	−
	E-00012551	MK751799	MK751808	MK751722	−
	E-00012552	MK751800	MK751809	MK751723	−
	GJO-0088904	MK751801	MK751810	MK751724	−
***Triblidium caliciiforme***	**HOU1053**	**MN519485**	**MN540636**	**MN538985**	**MN547962**
***Triblidium hubeiense***	**HOU1350A**	**MN541813**	**MN541811**	**MN541828**	**MN565260**
***Triblidium rostriforme***	**HOU851A**	**MN541815**	**MN541820**	**MN541821**	**MN565263**
	**HOU889**	**MN541822**	**MN541817**	**MN541839**	**MN565262**
***Triblidium yunnanense***	**HOU470A**	**MN541818**	**MN541819**	**MN541810**	**MN565259**
	**HOU1179**	**MN541814**	**MN541809**	**MN541816**	**MN565261**
	**HOU875A**	**MN541840**	**MN541828**	**MN541812**	**MN551099**
*Tryblidiopsis pinastri*	AFTOL-ID 1319	−	DQ470983	−	DQ470935

### Phylogenetic analysis

The sequences, used in this study, included 22 taxa for the ITS matrix, 32 taxa for the LSU matrix, 30 taxa for the mtSSU, and 11 taxa of RPB2. *Bisporella citrina* (Batsch) Korf & S.E. Carp. (Helotiales, Helotiaceae) and *Neofabraea malicorticis* (Cordley) H.S. Jacks. (Helotiales, Dermateaceae) were selected as outgroups. Maximum parsimony (MP) and Bayesian Inference (BI) analyses were performed on the concatenated ITS–LSU–mtSSU–RPB2 dataset. Each dataset was first aligned with Clustal X and then manually adjusted to allow maximum sequence similarity in Se-Al v.2.03a (Thompson et al. 1997; Rambaut 2000). Ambiguously aligned regions were excluded from the analysis by hand. Alignments were submitted to TreeBASE under accession number S25247. A partition homogeneity test was performed to determine the congruence of ITS, LSU, mtSSU and RPB2 (Farris et al. 1995; Huelsenbeck et al. 1996). After a positive outcome, the datasets were analysed together. The datasets were prepared and analysed with the maximum parsimony (MP) method using PAUP* 4.0b10 (Swofford 1998). The phylogenetic analysis was conducted using heuristic searches with 1000 replicates of random-addition sequence, tree bisection reconnection (TBR) branch swapping and no maxtree limit. All characters were equally weighted and unordered. Gaps were treated as missing data to minimise homology assumptions. A bootstrap analysis was performed with 1000 replicates, each with 100 random taxon addition sequences. Maxtrees were set to 1000 and TBR branch swapping was employed. For the Bayesian analysis, MrModeltest 2.3 with the Akaike Information Criterion (AIC) was used to choose the best-fit substitution models for the concatenated dataset: GTR+I+G for both ITS and LSU, HKY+I+G for mtSSU and SYM+G for RPB2. The Bayesian analysis was performed with MrBayes 3.1.2 (Huelsenbeck et al. 2011, Ronquist and Huelsenbeck 2003) with two sets of four chains (one cold and three heated) and the Stoprule option in effect, halting the analyses at an average standard deviation of split frequencies of 0.01. The sample frequency was set to 100 and the first 25% of trees were removed as burn-in and the remaining trees were kept and combined into one 50% majority-rule consensus tree. Bayesian Posterior Probabilities (PP) were obtained from the 50% majority consensus of the remaining trees. Clades receiving both bootstrap values of maximum parsimony (BP) ≥ 70% and PP ≥ 0.95 were considered to be significantly supported.

## Results

### Molecular phylogeny

**Figure 1.**
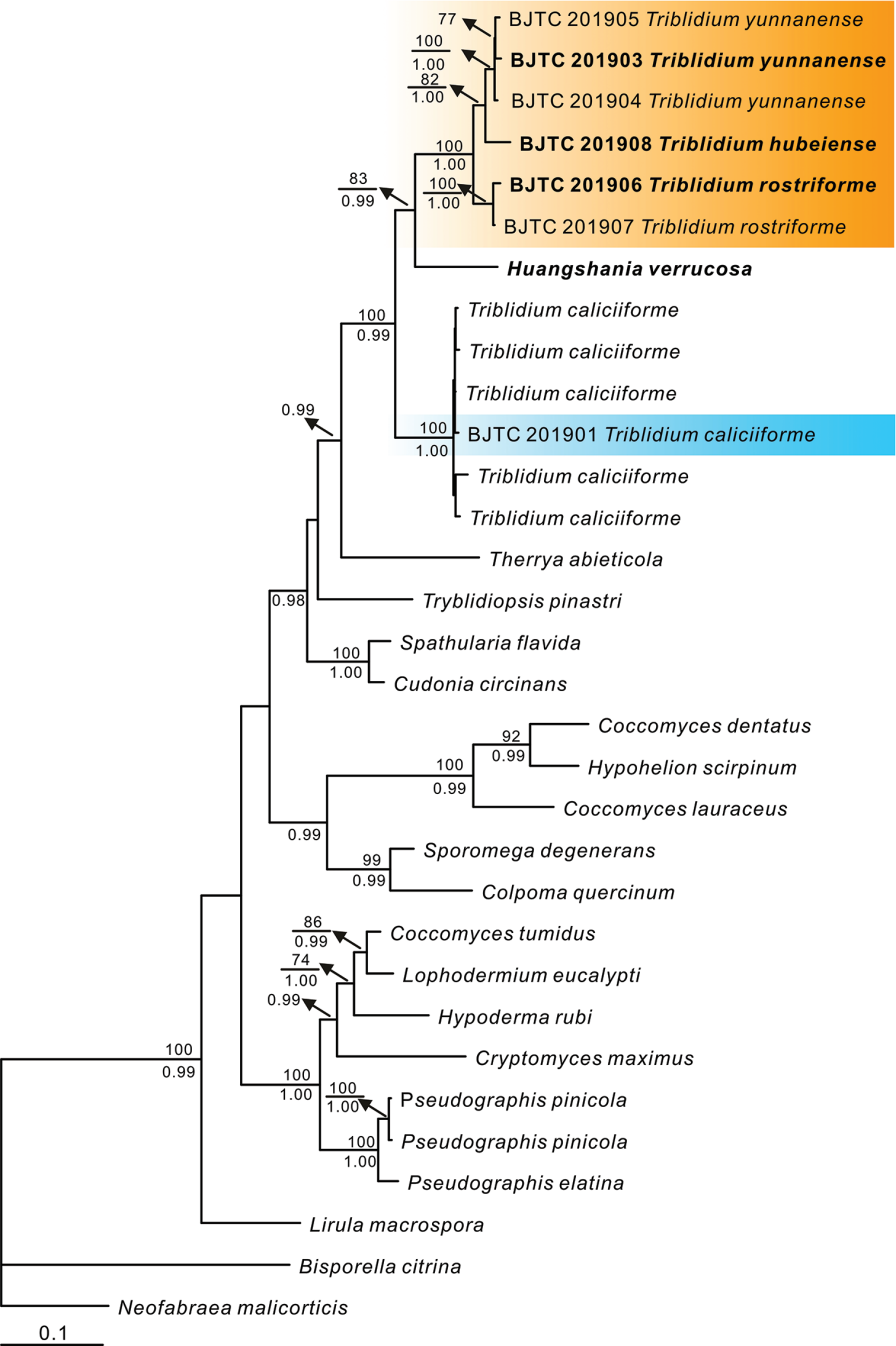
A phylogenetic tree generated by maximum parsimony and Bayesian analysis of the combined ITS, LSU, mtSSU and RPB2 sequences, using *B. citrina* and *N. malicorticis* as outgroups. Bootstrap values of maximum parsimony ≥ 70% are shown above the respective branches. Bayesian posterior probabilities ≥ 0.95 are marked below the branches. Sequences in bold indicate that the sequences are from the holotypes.

The phylogenetic analyses, based on the concatenated four-locus (ITS, LSU, mtSSU, RPB2) DNA matrix, included 32 taxa and 3472 characters, of which 843 were parsimony-informative. The maximum parsimony analysis resulted in one most parsimonious tree with a length (TL) of 2991 steps, consistency index (CI) of 0.697, retention index (RI) of 0.754 and homoplasy index (HI) of 0.303. Except for the two outgroup species, *B. citrina* and *N. malicorticis*, all the other taxa formed one highly supported clade. *Lirula macrospora* (R. Hartig) Darker resolved as sister to all the remaining taxa (Rhytismatales). This result is similar to the topology of Lantz et al. (2011). The Triblidiaceae samples formed a well-supported clade (BP = 100%, PP = 0.99; Fig. 1). The type species of *Huangshania*, *H. verrucosa* O.E. Erikss., is nested in the *Triblidium* clade (Fig. 1).

### Taxonomy

#### Triblidium hubeiense

Taxon classificationFungiTriblidialesTriblidiaceae

T. Lv & C. L. Hou sp. nov.

37265476-D614-51BB-A66E-8247FAE7D9D0

832358

Figs 2 , 3

##### Diagnosis.

Similar to *Triblidium sherwoodiae* but different by apically not swollen and unbranched paraphyses and homolateral curved ascospores, with a smaller L/W ratio of 1.4–2.3 (average ratio of 1.83) (average ratio of 2.52 for *T. sherwoodiae*).

**Holotype.** On dead twigs of *Rhododendron* sp., CHINA, Hubei Province, Shennongjia National Nature Reserve, 31.4360 N; 110.3014 E, alt. ca. 2900 m, 23 July 2018, C.-L. HOU1350A (BJTC 201908).

##### Description.

Ascomata erumpent from the bark, circular or rectangular in outline, 1.3–2.0 mm diam., solitary or occasionally confluent, with a black (#211414) outer surface that is sculptured with polygonal areolae, opening by irregular splits to expose a yellow (#ffc14f) hymenium. In median vertical section, ascomata 500–600 μm thick. Covering stroma 270–300 μm thick near the central part of ascomata, decreasing to 65–110 μm at the edge, consisting of an outer layer of highly melanized hyphae with a few remnants of host tissue embedded in the surface and an inner layer of hyaline hyphae. Basal layer 65–160 μm thick, composed of highly melanized hyphae with hyaline hyphae towards the internal matrix of stroma that is 75–125 μm thick, composed of textura intricata. Subhymenium 45–75 μm thick consisting of small, irregular textura angularis. Excipulum absent. Paraphyses 200–230 × ca. 1 μm, filiform, multi-guttulate, guttulae visible in water and IKI but disappearing in both lactophenol solution and 5% KOH, not swollen and branched at the apex, extending past mature asci. Asci ripening sequentially, 160–200 × 15–24 μm, cylindrical, thin-walled, without circumapical thickening, rounded at the apex, 6–8-spored. Ascospores 20–30 × 12–18 μm, L/W ratio of 1.4–2.3 (average ratio of 1.83), ellipsoidal, often curved homolateral, hyaline, at first aseptate, becoming muriform at maturity, with 6–8 transverse septa and a few longitudinal and oblique septa, without a gelatinous sheath, inamyloid in IKI.

**Figure 2.**
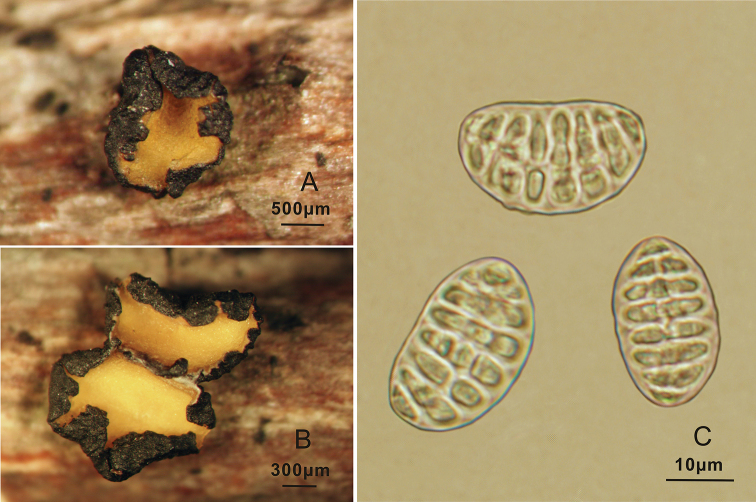
*Triblidium hubeiense* (Holotype, BJTC 201908) on *Rhododendron* sp. twig **A, B** mature dried ascomata observed under dissecting microscope **C** dead ascospores in water.

**Figure 3.**
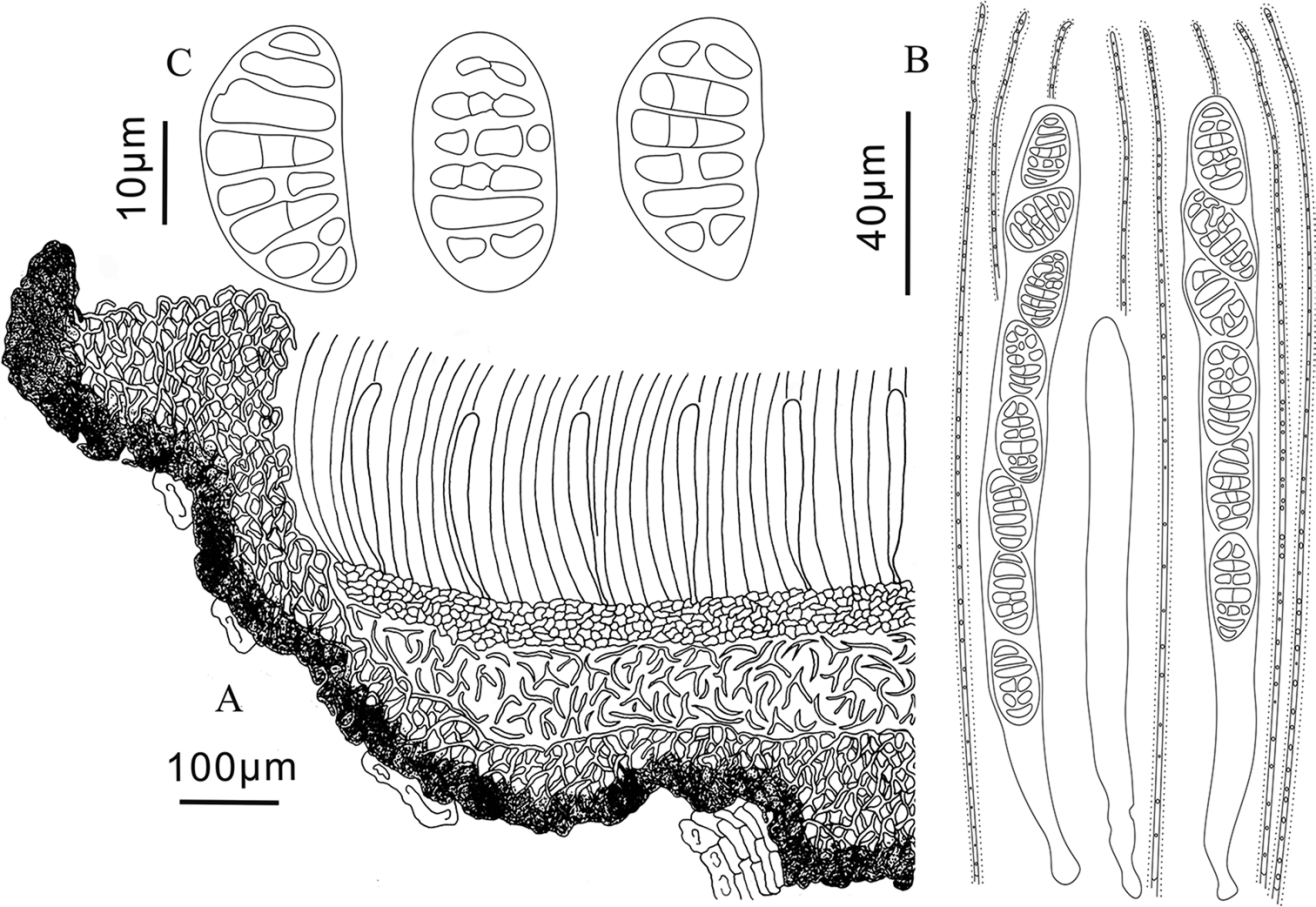
*Triblidium hubeiense* (Holotype, BJTC 201908) **A** ascoma in median vertical section **B** paraphyses, mature asci with ascospores and immature ascus **C** dead ascospores in water.

Conidiomata and zone lines not seen.

##### Known distribution.

Known from a single collection from Shennongjia National Nature Reserve, Hubei Province, China.

##### Etymology.

Referring to the Hubei Province where the specimen was collected.

##### Comments.

*Triblidium hubeiense* is similar to *T. sherwoodiae* Magnes and *T. carestiae* (De Not.) Rehm, but *T. sherwoodiae* has paraphyses with swollen terminal cell, straight ascospores and is only found on *Pinus ponderosa*; *T. carestiae* commonly has 3–8 ascospores per ascus, ascospores usually with beak-like structure at poles, 7–14 transverse septa and apically branched paraphyses.

#### Triblidium rostriforme

Taxon classificationFungiTriblidialesTriblidiaceae

T. Lv & C. L. Hou sp. nov.

9018C9C3-9F02-51D2-8DE7-10041742AA13

832359

Figs 4 , 5

##### Diagnosis.

Different from most *Triblidium* species by producing longer ascospores that have rostriform structures at the poles.

**Holotype.** On dead twigs of *Rhododendron* sp., CHINA, Yunnan Province, Lijiang, Laojunshan, 26.6831 N; 99.6997 E, alt. ca. 4056 m, 25 June 2011, C.-L. HOU 889 (BJTC 201906).

##### Description.

Ascomata erumpent from bark, elliptical in outline, 0.85–1.7 mm diam., solitary, with a black (#211414) outer surface that is sculptured with polygonal areolae, opening by irregular splits to expose the hymenium. In median vertical section, ascomata 350–550 μm thick. Covering stroma 45–70 μm thick, consisting of an outer layer of highly melanized hyphae with some host tissues incorporated into the surface and an inner layer of hyaline hyphae. Basal layer 40–80 μm thick, composed of a lower, highly melanized layer with hyaline hyphae towards the internal matrix of the stroma which is 40–98 μm thick, composed of textura intricata. Subhymenium 25–45 μm thick, consisting of hyaline textura angularis. Excipulum moderately developed, formed by marginal paraphyses. Paraphyses 180–240 × ca. 1 μm, filiform, occasionally branched, sparsely guttulate, guttulae visible in water and IKI but disappearing in both lactophenol solution and 5% KOH. Asci ripening sequentially, 160–220 × 15–25 μm, cylindrical, thin-walled, without circumapical thickening, rounded at the apex, 8-spored. Ascospores 35–50 × 12–20 μm, L/W ratio of 2.0–3.8 (average ratio of 2.55), elliptical, with rostriform structures at the poles, hyaline, at first aseptate, becoming muriform at maturity, with usually 6 transverse septa and a few longitudinal and oblique septa, without gelatinous sheath, inamyloid in IKI.

**Figure 4.**
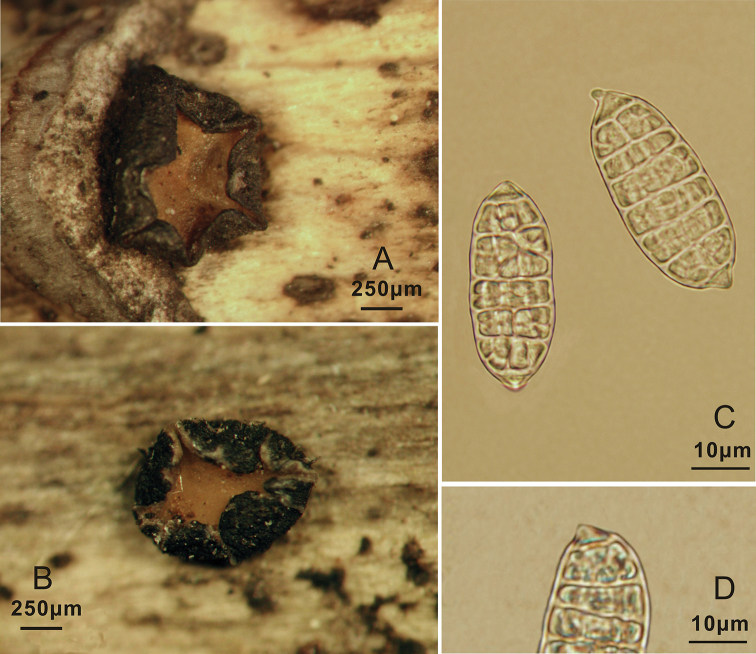
*Triblidium rostriforme* (Holotype, BJTC 201906) on *Rhododendron* sp. twig **A, B** mature dried ascomata observed under a dissecting microscope **C** dead ascospores in water **D** rostriform structure of ascospores.

**Figure 5.**
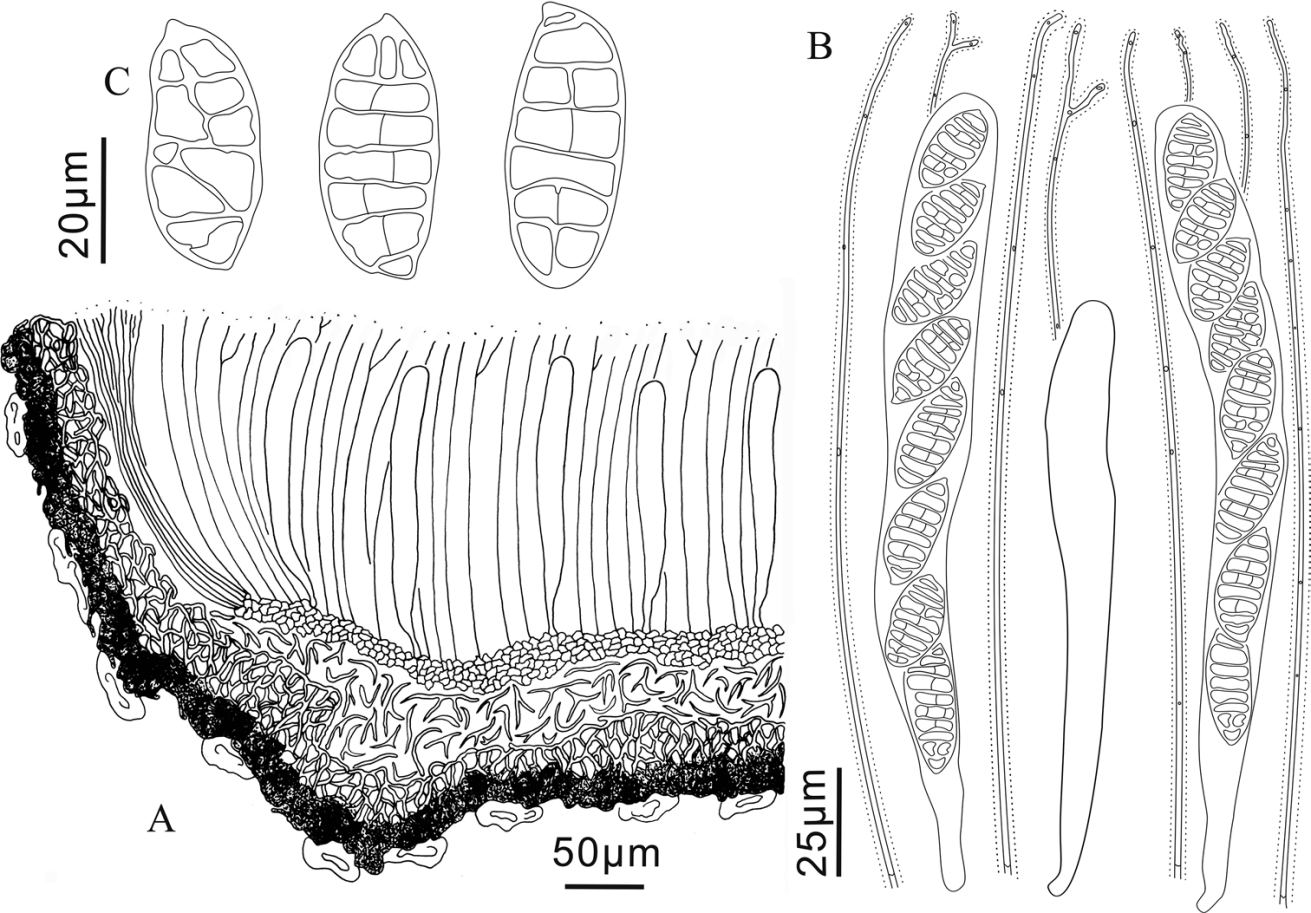
*Triblidium rostriforme* (Holotype, BJTC 201906) **A** ascoma in median vertical section **B** paraphyses, mature asci with ascospores and immature ascus **C** dead ascospores in water.

Conidiomata and zone lines not seen.

##### Etymology.

From Latin, *rostriforme*, referring to the beak-like protrusions observed at the ascospore poles.

##### Additional specimen examined.

On dead twigs of *Rhododendron* sp., CHINA, Yunnan Province, Lijiang, Laojunshan, 26.6702 N; 99.7002 E, alt. ca. 4110 m, 24 June 2011, C.-L. HOU 851A (BJTC 201907).

##### Comments.

*Triblidium rostriforme* is similar to *T. carestiae* (De Not.) Rehm but *T. carestiae* commonly has asci with 3–8 ascospores, ascospores with usually 7–14 transverse septa and ramose, multi-guttulate paraphyses.

#### Triblidium yunnanense

Taxon classificationFungiTriblidialesTriblidiaceae

T. Lv & C. L. Hou sp. nov.

90911E23-082C-5EC0-A251-E011985AA5CA

832360

Figs 6 , 7

##### Diagnosis.

Different from *T. hafellneri* by its ascospores with 6–8 transverse septa, narrow asci and geographical range. Different from its phylogenetically closest relatives (*T. hubeiense* and *T. rostriforme*) by the size and the shape of ascomata and ascospores.

**Holotype.** On twigs of *Rhododendron* sp., CHINA, Yunnan Province, Lijiang, Laojunshan, 26.6571 N; 99.6944 E, alt. ca. 4070 m, 25 June 2011, C.-L. HOU 875A (BJTC 201903).

##### Description.

Ascomata erumpent from bark, circular or slightly irregular in outline, 0.5–0.8 mm diam., solitary, with a black (#211414) outer surface that is sculptured with polygonal areolae, opening by irregular splits to expose the hymenium. In median vertical section, ascomata 300–400 μm thick. Covering stroma 45–75 μm, consisting of an outer layer of highly melanized hyphae with remnants of host tissue incorporated into the outer surface and an inner layer of hyaline hyphae. Basal layer 45–88 μm thick, composed of an outer layer of highly melanized hyphae and short, thick, hyaline hyphae towards the internal matrix of stroma that is 60–85 μm thick, composed of thick hyphae. Subhymenium 35–59 μm thick, consisting of hyaline textura angularis. Excipulum 25–35 μm thick, formed by marginal paraphyses. Paraphyses 180–230 × 1–1.2 μm, filiform, often branched, multi-guttulate, guttulae visible in water and IKI but disappearing in both lactophenol solution and 5% KOH. Asci ripening sequentially, 150–200 × 13–18 μm, cylindrical, thin-walled, without circumapical thickening, rounded at the apex, 6–8-spored. Ascospores 20–30 × 10–15 μm, L/W ratio of 1.7–2.5 (average ratio of 1.99), ellipsoid, hyaline, at first aseptate, becoming muriform at maturity, with usually 6–8 transverse septa and a few longitudinal and oblique septa, without gelatinous sheath, inamyloid in IKI.

**Figure 6.**
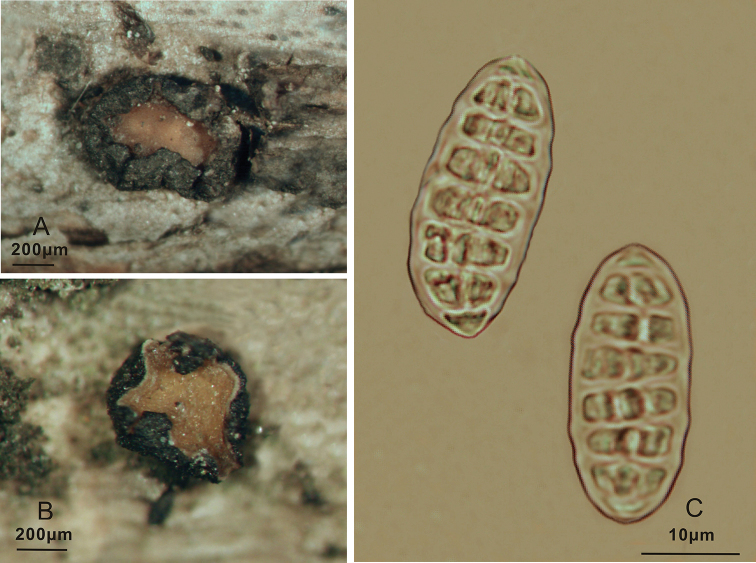
*Triblidium yunnanense* (Holotype, BJTC 201903) on *Rhododendron* sp. twig **A, B** mature dried ascomata observed under a dissecting microscope **C** dead ascospores in water.

**Figure 7.**
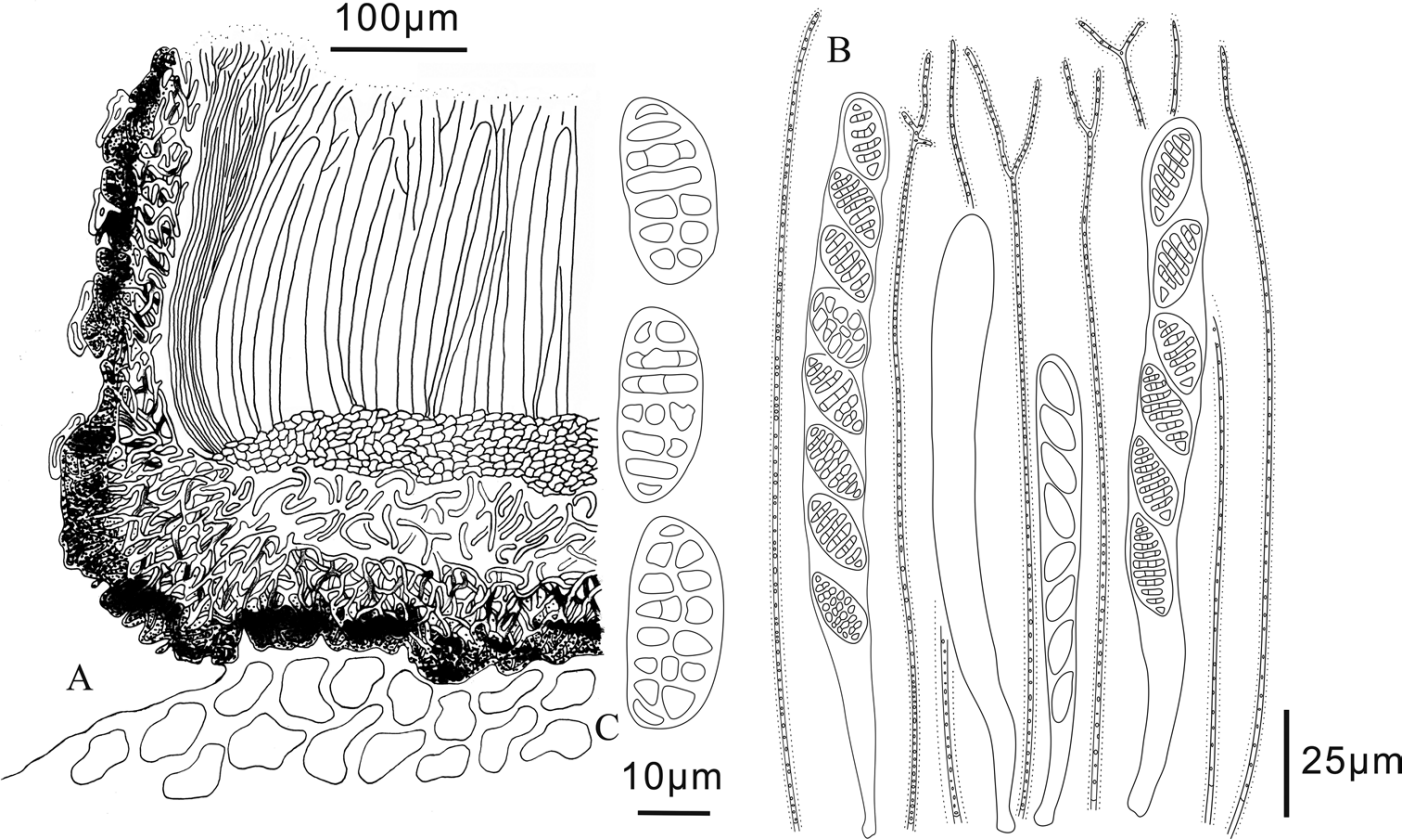
*Triblidium yunnanense* (Holotype, BJTC 201903) **A** ascoma in median vertical section **B** paraphyses, mature asci with ascospores and immature ascus **C** dead ascospores in water.

Conidiomata and zone lines not seen.

##### Etymology.

Referring to the Yunnan Province where the holotype specimens were collected.

##### Additional specimens examined.

On twigs of *Rhododendron* sp., CHINA, Yunnan Province, Lijiang, Laojunshan, 26.6741 N; 99.6930 E, alt. ca. 4040 m, 11 July 2007, C.-L. HOU 470A (BJTC 201904). On dead twigs of *Rhododendron* sp., CHINA, Sichuan Province, Mt. Emeishan, 29.5185 N; 103.3329 E, alt. ca. 3010 m, 12 July 2014, C.-L. HOU 1179 (BJTC 201905).

##### Comments.

*Triblidium yunnanense* is similar to *T. hafellneri* Magnes, but the latter has asci 20–25 μm wide, ascospores with 7 transverse septa and occurs on *Vaccinum ovatum*, *Calluna vuglaris*, *Salix* spp., and *Nothofagus antarctica* in Europe and the Americas. *Triblidium yunnanense* has a close relationship to the two other new species in this study, but *T. rostriforme* has larger ascomata, ascospores with special beak-like structures and *T. hubeiense* has larger ascomata, unbranched paraphyses, a moderately developed excipulum, a thicker covering stroma, basal layer and subhymenium.

#### Triblidium verrucosum

Taxon classificationFungiTriblidialesTriblidiaceae

(O.E. Erikss.) T. Lv, C. L. Hou & P. R. Johnst. comb. nov.

09CEDD9B-BA96-5A57-A3DB-B76CC82A3020

832361

≡ Huangshania verrucosa O.E. Erikss., Systema Ascomycetum 11: 2, 1992.

##### Notes.

The placement of this species in *Triblidium* is demonstrated by the phylogeny presented in Fig. 1. Eriksson (1992) discussed the similarities between *Huangshania* and *Triblidium* in macro-morphology and in the morphology of hamathecial tissues and asci. The two genera differed only in ascospore morphology (elongate-phragmosporous vs. ellipsoidal-muriform). Karakehian et al. (2019) reviewed that ascospore morphology is a poor predictor of relatedness amongst these fungi. *Huangshania verrucosa* is the type species of the genus, therefore, *Huangshania*, is synonymized here under *Triblidium*.

## Discussion

The morphological characteristics of the species described here are typical of *Triblidium* (Magnes 1997): ascomata on twigs of *Rhododendron* spp., muriform, inamyloid ascospores, and highly melanized ascomata with roughened outer surfaces. Based on our molecular phylogenetic analyses (Fig. 1), the three newly described species form a highly supported clade, sister to *T. verrucosum*. *Triblidium yunnanense* and *T. hubeiense* form a well-supported subclade sister to *T. rostriforme*. The similarity of ITS amongst these three new species is 90–95%. The sequences generated from the specimens of *T. caliciiforme* collected from France on bark of *Quercus* sp., clustered well with other sequences accepted as *T. caliciiforme* by Karakehian et al. (2019).

The strongly supported phylogenetic relationship justifying the synonymy of *Huangshania* with *Triblidium* was not detected by Karakehian et al. (2019) because only the type species of *Triblidium* had DNA sequences available. If *Huangshania* is not placed in synonymy, the addition of the new Chinese *Triblidium* species described here would result in *Triblidium* being paraphyletic. The alternative solution, to erect a new genus for the Chinese species, has no morphological support, since these species are very similar to *T. caliciiforme* in both morphology and ecology. In 1992, Eriksson erected *Huangshania* as a genus only according to the morphology of the spores. Karakehian et al. (2019) examined the ascospores of *H. verrucosa* and noted that ascospore morphology appears to be a poor predictor of phylogenetic relationships amongst these fungi. It is worth noting that the rostrum of the ascospores in *T. rostriforme* and *T. carestiae* bear some similarity to the plug-like appendages of *H. verrucosa*. Furthermore, we did not transfer *H. novae-fundlandiae* (Rehm) Magnes, another species in *Huangshania*, to *Triblidium* since sequences were lacking.

In conclusion, three new *Triblidium* species from China were described in detail by both morphological and phylogenetic analyses. The new species, discovered in China, illustrate that these fungi are more widespread than previously known. Sequences from these new collections have expanded the representation of this genus in NCBI GenBank and helped our understanding of the family Triblidiaceae. *Huangshania* is placed in synonymy with *Triblidium* in order to maintain its monophyly, further demonstrating that ascospore morphology alone may be a poor predictor of evolutionary relationships.

### Key to species of *Triblidium*

**Table:** 

1	Ascospores phragmosporous	***T. verrucosum***
–	Ascospores muriform	**2**
2	Ascospores ellipsoid, without rostriform beaks at the poles	**3**
–	Ascospores ellipsoid with rostriform beaks at the poles	**4**
3	Ascomata ≥1mm diam	**5**
–	Ascomata <1mm diam	**6**
4	Paraphyses multi-guttulate, often branched at the apex; thick-walled asci with 3–8 ascospores; ascospores with 7–14 transverse septa	***T. carestiae***
–	Paraphyses sparsely guttulate, occasionally branched at the apex; asci thin-walled with 8-ascospores; ascospores with 6 transverse septa	***T. rostriforme***
5	Asci 20–25 μm wide; ascospores with 7 transverse septa; occurring on *Vaccinum ovatum*, *Calluna vuglaris*, *Salix* spp. *and Nothofagus antarctica*	***T. hafellneri***
–	Asci 13–18 μm wide; ascospores with 6–8 transverse septa; only found on *Rhododendron* sp.	***T. yunnanense***
6	Occurring mainly on *Fagaceae* spp. and *Pinus* spp.	**7**
–	Occurring mainly on *Rhododendron* spp., asci 160–200 × 15–24 μm, ascospores 20–30 × 12–18 μm	***T. hubeiense***
7	Asci 230–280 × 25–30 μm, ascospores 30–48 × 12–20 μm	***T. caliciiforme***
–	Asci 150–190 × 13–23 μm, ascospores 28–35 × 11–14 μm	***T. sherwoodiae***

## Supplementary Material

XML Treatment for Triblidium hubeiense

XML Treatment for Triblidium rostriforme

XML Treatment for Triblidium yunnanense

XML Treatment for Triblidium verrucosum

## References

[B1] Eriksson OE (1992) *Huangshania verrucosa* gen. et spec. nov. (*Triblidiaceae*, *Triblidiales* ordo nov.) a discomycete on *Pinus* from China. Systema Ascomycetum 11(1): 1−10.

[B2] Farris JS Kallersjø M Kluge AG Bult C (1995) Testing significance of incongruence. Cladistics 10: 315−319. 10.1111/j.1096-0031.1994.tb00181.x

[B3] Gardes M Bruns TD (1993) ITS primers with enhanced specificity for basidiomycetes-application to the identification of mycorrhizae and rusts. Molecular Ecology 2: 113–118. 10.1111/j.1365-294X.1993.tb00005.x 8180733

[B4] Hou CL Li L Piepenbring M (2009) *Lophodermium pini-mugonis* sp. nov. on needles of *Pinus mugo* from the Alps based on morphological and molecular data. Mycological Progress 8: 29−33. 10.1007/s11557-008-0575-z

[B5] Huelsenbeck JP Bull JJ Cunningham CW (1996) Combining data in phylogenetic analysis. Tree 11: 152−158. 10.1016/0169-5347(96)10006-9 21237790

[B6] Huelsenbeck JP Ronquist F Nielsen R Bollback JP (2011) Bayesian inference of phylogeny and its impact on evolutionary biology. Science 294: 2310−2314. 10.1126/science.1065889 11743192

[B7] Johnston PR Quijada L Smith CA Baral H-O Hosoya T Baschien C Pärtel K Zhuang K-Y Haelewaters D Park D Carl S López-Giráldez F Wang Z Townsend JP (2019) A multigene phylogeny toward a new phylogenetic classification of Leotiomycetes. IMA Fungus 10(1): 1–22. 10.1186/s43008-019-0002-x PMC7325659 32647610

[B8] Karakehian JM Quijada L Friebes G Tanney JB Pfister DH (2019) Placement of *Triblidiaceae* in *Rhytismatales* and comments on unique ascospore morphologies in *Leotiomycetes* (Fungi, *Ascomycota*). Mycokeys 54: 99–133. 10.3897/mycokeys.54.35697 31258376 PMC6592975

[B9] Liu YJ Whelen S Hall BD (1999) Phylogenetic relationships among *Ascomycetes*: evidence from an RNA polymerse II subunit. Molecular Biology and Evolution 16: 1799–1808. 10.1093/oxfordjournals.molbev.a026092 10605121

[B10] Lantz H Johnston PR Park D Minter DW (2011) Molecular phylogeny reveals a core clade of *Rhytismatales* Mycologia 103: 57−74. 10.3852/10-060 20943536

[B11] Magnes M (1997) Weltmonographie der *Triblidiaceae*. Bibliotheca Mycologica 165: 1−177.

[B12] Prieto M Schultz M Olariaga L Wedin M (2019) Lichinodium is a new lichenized lineage in the Leotiomycetes. Fungal Diversity 94: 23–39. 10.1007/s13225-018-0417-5

[B13] Rambaut A (2000) Estimating the rate of molecular evolution: incorporating non-contemporaneous sequences into maximum likelihood phylogenies. Bioinformatics 16: 395−399. 10.1093/bioinformatics/16.4.395 10869038

[B14] Rehm H (1888−1896) Die Pilze Deutschlands, Oesterreichs und der Schweiz. III. Abtheilung: Ascomyceten: Hysteriaceae und Discomyceten. Dr. L. Rabenhorst’s Kryptogamen-Flora von Deutschland, Oesterreich und der Schweiz (2^nd^ edn.) Bd 1: Abt. 3. Leipzig, Verlag von Eduard Kummer, 1275 pp 10.5962/bhl.title.1356

[B15] Rehm H (1912) Zur Kenntnis der Discomyceten Deutschlands, Deutsch-Österreichs und der Schweiz. Berichte der Bayerischen Botanischen Gesellschaft zur Erforschung der Heimischen Flora 13: 102–206.

[B16] Rehner SA Samuels GJ (1994) Taxonomy and phylogeny of Gliocladium analysed from nuclear large subunit ribosomal DNA sequences. Mycological Research 98(6): 625–634. 10.1016/S0953-7562(09)80409-7

[B17] Ronquist F Huelsenbeck JP (2003) MrBayes 3: Bayesian Phylogenetic inference under mixed models. Bioinformatics 19: 1572−1574. 10.1093/bioinformatics/btg180 12912839

[B18] Swofford DL (1998) PAUP*: phylogenetic analysis using parsimony (and other methods). Version 4. Sinauer Associates, Sunderland.

[B19] Thompson JD Gibson TJ Plewniak F Jeanmougin F Higgins DG (1997) The ClustalX windows interface: flexible strategies for multiple sequence alignment aided by quality analysis tools. Nucleic Acids Research 24: 4876−4882. 10.1093/nar/25.24.4876 PMC147148 9396791

[B20] Vilgalys R Hester M (1990) Rapid genetic identification and mapping of enzymatically amplified ribosomal DNA from several Cryptococcus species. Journal of Bacteriology 172: 4238–4246. 10.1128/jb.172.8.4238-4246.1990 2376561 PMC213247

[B21] White TJ Bruns TD Lee S Taylor J (1990) Amplification and direct sequencing of fungal ribosomal RNA genes for phylogenetics. In: Innis MA Gelfand DH Sninsky JJ White TJ (Eds) PCR Protocols, a Guide to Methods and Applications.Academic Press, San Diego, 315–322. 10.1016/B978-0-12-372180-8.50042-1

[B22] Zoller S Scheidegger C Sperisen C (1999) PCR primers for the amplification of mitochondrial small subunit ribosomal DNA of lichen-forming ascomycetes Lichenologist 31: 511−516. 10.1006/lich.1999.0220

